# Direct intercellular communications dominate the interaction between adipose-derived MSCs and myofibroblasts against cardiac fibrosis

**DOI:** 10.1007/s13238-015-0196-7

**Published:** 2015-08-14

**Authors:** Xiaokang Li, Hui Zhao, Chunxiao Qi, Yang Zeng, Feng Xu, Yanan Du

**Affiliations:** Department of Biomedical Engineering, School of Medicine, Collaborative Innovation Center for Diagnosis and Treament of Infectious Diseases, Tsinghua University, Beijing, 100084 China; School of Life Sciences, Tsinghua University, Beijing, 100084 China; MOE Key Laboratory of Biomedical Information Engineering, School of Life Science and Technology, Xi’an Jiaotong University, Xi’an, 710049 China; Bioinspired Engineering and Biomechanics Center (BEBC), Xi’an Jiao Tong University, Xi’an, 710049 China

**Keywords:** cardiac fibrosis, stem cell therapy, adipose-derived mesenchymal stem cells, myofibroblasts, cell-to-cell contact, anti-fibrosis

## Abstract

The onset of cardiac fibrosis post myocardial infarction greatly impairs the function of heart. Recent advances of cell transplantation showed great benefits to restore myocardial function, among which the mesenchymal stem cells (MSCs) has gained much attention. However, the underlying cellular mechanisms of MSC therapy are still not fully understood. Although paracrine effects of MSCs on residual cardiomyocytes have been discussed, the amelioration of fibrosis was rarely studied as the hostile environment cannot support the survival of most cell populations and impairs the diffusion of soluble factors. Here in order to decipher the potential mechanism of MSC therapy for cardiac fibrosis, we investigated the interplay between MSCs and cardiac myofibroblasts (mFBs) using interactive co-culture method, with comparison to paracrine approaches, namely treatment by MSC conditioned medium and gap co-culture method. Various fibrotic features of mFBs were analyzed and the most prominent anti-fibrosis effects were always obtained using direct co-culture that allowed cell-to-cell contacts. Hepatocyte growth factor (HGF), a well-known anti-fibrosis factor, was demonstrated to be a major contributor for MSCs’ anti-fibrosis function. Moreover, physical contacts and tube-like structures between MSCs and mFBs were observed by live cell imaging and TEM which demonstrate the direct cellular interactions.

**Electronic supplementary material:**

The online version of this article (doi:10.1007/s13238-015-0196-7) contains supplementary material, which is available to authorized users.

## INTRODUCTION

Myocardial infarction accounts for over 40% of cardiovascular diseases (CVD) related human mortality, as reported by the World Health Organization (WHO, 2013). Cardiac fibrosis, the most eminent pathological feature post infarction (van den Borne et al., 2010; Fan et al., 2012; Weber et al., 2013), would form a scattering area of fibrosis scars that generate tonic contraction forces and interfere with the original conduction property of healthy myocardium, thus greatly impairs the physiological functions (i.e. blood pumping) of normal heart (Rohr, 2009; Weber et al., 2013). Histological characterization revealed that an excessive deposition of fibrillar collagen was accumulated within the infarcted myocardium and an altered cell population which was positive for alpha-smooth muscle actin (α-SMA) replaced the lost cardiomyocytes, namely myofibroblasts (mFBs) (Rohr, 2009). mFBs were believed to mainly originate from interstitial cardiac fibroblasts (Brown et al., 2005; Czubryt, 2012) which are responsible for the stability of extracellular matrix in heart and its dynamic balance with cardiomyocytes (Fan et al., 2012; Weber et al., 2013). The transition from interstitial fibroblasts to mFBs has been demonstrated to be initiated by transforming growth factor-beta 1 (TGF-β1) secreted by both immunocytes and necrotic cardiomyocytes (Brown et al., 2005; Rohr, 2009; Czubryt, 2012; Weber et al., 2013).

The current treatments for myocardial infarction, including the administration of thrombolytic drugs (e.g. streptokinase, urokinase and alteplase) (Tomasevic et al., 2008; Minami et al., 2010; Sehestedt et al., 2011; Lyngbaek et al., 2012; Juarez-Herrera and Jerjes-Sanchez, 2013; Lippi et al., 2013), the implantation of vascular stents (Armstrong, 2006; Stefanini and Windecker, 2012; Tokushige et al., 2013; Zhang et al., 2013) and bypass operation (Favaloro, 1969, 1971) mainly concentrated on improving the hemodynamics thus restoring the blood supply to infarcted myocardium. Nonetheless, the efficacy of these conventional therapies was limited as they could hardly compensate for the massive loss of necrotic cardiomyocytes, which account for approximately 25% of total cardiomyocytes during a single episode of infarction (Deutsch et al., 2013). Given the limited regenerative capacity of adult human heart, effective therapies should focus on the remuscularization of the diseased heart (Deutsch et al., 2013). Therefore, transplantation of autologous cells into the diseased heart has shown to be a reasonable and effective therapeutic strategy. During the past decade, multiple cell types, including endothelial progenitors (Asahara et al., 1997) and mesenchymal stem cells (MSCs, either derived from bone marrow or adipose tissue) (Miyahara et al., 2006; Emmert et al., 2013; Blocki et al., 2015; Li and Zhang, 2015) have shown therapeutic effects (shrunken area of fibrotic scar and thickened ventricle wall post-infarction) on cardiac fibrosis, among which the utility of MSCs has reached phase II or III clinical trials due to their ease of access and consistent outcome (Sheridan, 2013). However, the exact behavior of MSCs post-transplantation *in vivo* was barely known and the exact underlying cellular mechanisms were not fully understood. Although the development of advanced imaging techniques, e.g. magnetic resonance imaging (MRI) has been used to track the stem cells post transplantation (Drey et al., 2013; Emmert et al., 2013), the intercellular activity between MSCs and host tissue cells remained difficult to monitor. In addition, the paracrine activity of MSCs was reported to activate the quiescent cardiac progenitor cells or stimulate the residual cardiomyocytes to re-enter proliferative phase (Ranganath et al., 2012). However, few of these studies have provided solid explanation on how MSCs attenuate the fibrosis condition within the infarcted myocardium, since the hostile avascular and hypoxic environment could not support cell survival and factor diffusion to help remuscularization of the diseased heart.

As the main ‘architect’ of cardiac fibrosis, the interplay between cardiac mFBs and MSCs should be investigated to provide a better understanding for cardiac fibrosis therapy. Some studies showed that the conditioned medium from MSC culture (Ohnishi et al., 2007; Mias et al., 2009; Wang et al., 2011; Mao et al., 2013) could inhibit the proliferation, the expression of α-SMA and collagen production of mFBs, and the secretion of matrix metalloproteinases (MMPs) by fibroblasts was elevated as well. Nonetheless, the treatment of conditioned medium *in vitro* cannot fully recreate the condition of cell therapy *in vivo* where multiple intercellular activities were involved. For instance, Cselenyák et al. observed a significant dependency of cell-to-cell contact for MSC therapy to rescue the cardiomyocytes from cell death using an *in vitro* ischemia model, whereas MSCs cultured in inserts, the conventional format for paracrine studies, cannot exert similar beneficial effect (Cselenyak et al., 2010). Besides, Plotnikov et al. discovered that specific cellular contacts, namely nanotubes, were formed within the co-culture of MSCs with cardiomyocytes (Plotnikov et al., 2008). Therefore, the direct cell-to-cell contact, or so-called intercellular communication, would be a dominant factor for proper therapeutic benefit of MSCs to cardiac fibrosis.

To elucidate the dominant role of intercellular communications for MSC therapy, the direct cell co-culture model using adipose-derived MSCs and cardiac mFBs was introduced *in vitro* and multiple pathological features of mFBs, including cellular viability, biomarker expression (α-SMA, collagen), cellular contractility and motility were analyzed in the model, which were compared to the treatment of MSC conditioned medium in parallel. An engineered microfluidic-based co-culture platform was fabricated to further assess the importance of direct cell-to-cell communication for MSCs’ anti-fibrotic therapy. The system comprised two chambers separated by varying distances of cell-repellent gap, which would only allow communication by paracrine factors and no direct contact could be formed.

## RESULTS AND DISCUSSION

### Viability of mFBs during co-culture and comparison with treatment using MSC conditioned medium

**Figure 1:**
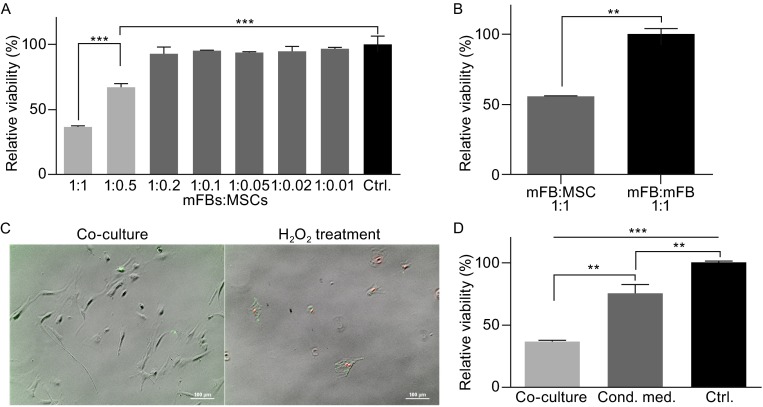
**Reduced viability of mFBs in MSC co-cultures with comparison to mFB monoculture and treatment by MSC conditioned medium**. (A) mFBs were co-cultured with MSCs (mitosis inactivated) in gradient ratios ranging from 1:0.01 to 1:1. All values were normalized to that of control group (mFB:MSC = 1:0, monocultured mFBs). (B) A group was set by replacing MSCs with same number of inactivated mFBs in order to eliminate the impact of high cell density on cell viability. (C) Cell apoptosis detection was performed using Annexin V/PI staining. Apoptotic cells (treated with H_2_O_2_) were stained green on membrane (Annexin V-FITC) and red in nuclei (propidium iodide, PI), while few apoptosis was detected in co-cultures. (D) Conditioned medium collected from normal MSC cultures were applied to mFBs and the resulting viability was compared to that of direct co-culture. Scale bar = 100 μm. Significance was defined as ‘**’ when *P* < 0.01 and ‘***’ when *P* < 0.001

The viability of mFBs was inhibited by co-culturing with MSCs in a dose-dependent manner (Fig. 1A). Comparable level of viability to control group was obtained when MSCs were administrated in low dosages (1%~20% of mFBs), while a significant decrease could be observed at high doses (half or the same as the number of mFBs). In order to eliminate the impact of contact inhibition on the reduced viability resulted from high cell density, MSCs in 1:1 co-culture were replaced by an equal number of mFBs, yet an obvious difference was still obtained (Fig. 1B), indicating the independence of reduced viability of mFBs on high cell densities. Annexin V/PI staining was also performed to verify whether the decreased viability of mFBs was caused by cell apoptosis. In addition, specificity control for the staining was included using cells treated with 500 nmol/L H_2_O_2_ solution. Apoptotic cells were stained green on membrane and red in nuclei, while few cells in co-culture were positive for both signals (Fig. 1C). Thus the ratio of 1:1 was selected for the following experiments.

Conditioned medium harvested from normal MSC cultures was used to incubate mFBs for 48 h as a conventional paracrine method, resulting in decreased viability of mFBs relative to controls (Fig. 1D). However, the viability reduction was not comparable to direct co-culture manner, indicating that specific stimuli from microenvironment (cell-to-cell communications) would be important for MSCs exerting better therapeutic effect. Indeed, it has been reported that preconditioning for MSCs would serve as a stress environment and enable better regenerative effects in various diseases (Haque et al., 2013). For example, the application of hypoxic preconditioning during culture would enhance the survival rate and therapeutic potential of MSCs in treating brain (Chang et al., 2013) or liver (Yu et al., 2013) injuries. Here mFBs culture recapitulated the cardiac fibrosis environment and thus served as the stress preconditioning for MSCs.

### Phenotypic and functional analysis of mFBs during co-culture

**Figure 2:**
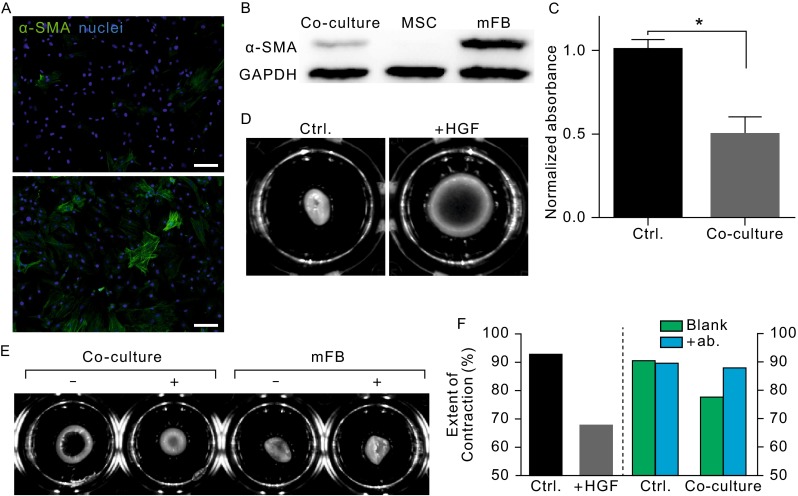
**Phenotypic characterization and function analysis of mFBs**. The expression of α-SMA, the most specific biomarker of mFBs compared to fibroblasts, was characterized by immunofluorescence staining (A, top: co-culture; bottom: control) and Western blotting (B). Green stained cells were regarded as mFBs and non-stained were MSCs. (C) Collagen production capacity of mFBs was measured by reading the absorbance of Sirius red labeled protein specimens. (D–F) The contractility of mFBs was measured by collagen contraction assay. HGF was supplemented to mFB-populated collagen gel lattice and the resulting area was compared with control (D). (E) Antibody against HGF was added to block the factors secreted by MSCs which was marked by ‘+’. (F) The quantitative analysis of contraction extents calculated by normalizing the lattice area to well size. Scale bar = 100 μm

Since the viability of mFBs was reduced in co-culture unrelated to apoptosis or contact inhibition, it was conjectured that the cellular phenotype of mFBs would alter to a more dormant state, which could be quiescent fibroblasts (Brown et al., 2005; Deutsch et al., 2013; Weber et al., 2013). The expression of α-SMA, which has been a typical marker for distinguishing fibroblasts from active mFBs, was assayed in co-cultures. Immunofluorescence images displayed faint expression profile for mFBs in co-culture compared to those in normal cultures (Fig. 2A, with the same total cell number). Further verification was accomplished by Western blotting of α-SMA (Fig. 2B), where it was shown that MSCs hardly expressed α-SMA compared to normal mFBs. As mFBs were capable of producing massive extracellular matrix proteins, most of which was composed of collagen, the total collagen content was quantified for both co-culture and control group. Collagen was labelled with Sirius Red dye and extracted from cultures. Similar to the decreased expression of α-SMA, the production of collagen was significantly reduced in co-culture (Fig. 2C). It therefore was assumed that mFBs would undergo dedifferentiation process towards fibroblasts, a quiescent cell population, during co-culture with MSCs.

Besides the phenotypic alteration of cells, mFBs could also exert strong contractile ability which could be characterized by the extent of collagen gel contraction *in vitro*. Gels populated by co-cultured mFBs remained relatively larger in size compared with those populated with pure mFBs (Fig. 2E, marked by ‘-’), and mFB-populated collagen gels would contract independently of different comprising number of mFBs (data not shown), implying that the contractile property of mFBs was greatly inhibited by MSCs. As a member of MSC secretome, HGF has been demonstrated to be an effective inhibitor for fibrosis condition. Collagen gel lattice remained much bigger in size than control group with the addition of exogenous HGF (Fig. 2D), and the HGF antibody could block the inhibitory effect of MSCs in co-culture without affecting the original contractile ability of mFBs (Fig. 2E).

Therefore, it was demonstrated here that co-culturing with MSCs could drive the mFBs return to a quiescent dedifferentiated state where some of pathological characteristics (both phenotypic and functional), e.g. α-SMA expression and collagen gel contraction ability, were significantly alleviated. HGF played an important role for enhancing the anti-fibrosis activity of MSCs. Moreover, MSCs themselves were reported to have strong contractile ability in mono-culture (Sumanasinghe et al., 2009; Espagnolle et al., 2014) and similar results were also obtained in this study (Fig. S1), implying that specific microenvironment was essential to stimulate the therapeutic potential of MSCs.

### Comparison of mFBs dedifferentiation during co-culture between direct contact mode and gap mode

**Figure 3:**
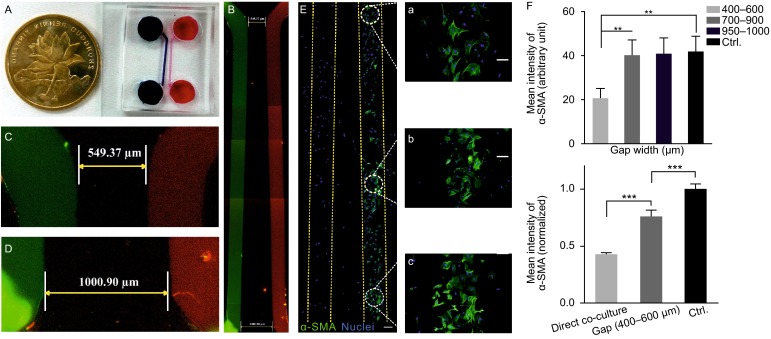
**The microfluidic device for MSC/mFB co-culture in gap mode**. (A) Macro-size demonstration of the device compared with a 50 cent coin (25 mm diameter). (B) The culture chambers within the device were highlighted by red (Dylight 594-conjugated IgG) and green (AlexaFluor 488-conjugated IgG) stains, respectively. The narrowest width between chambers was ~500 μm (C) while the widest ~1000 μm (D). MSCs and mFBs were seeded into different chambers (E, Scale bar = 100 μm). The α-SMA expression of mFBs was characterized by measuring the fluorescence intensity under constant image settings. Cells growing with varying gap width (a: 400–600 μm; b: 700–900 μm; c: 950–1000 μm; scale bar = 100 μm) were analyzed. (F) Fluorescence intensities were compared among mFBs residing on different gap widths (upper graph), or between directly co-cultured mFBs and mFBs with the narrowest gap (bottom graph)

An engineered gap mode co-culture device was fabricated using PDMS (Fig. 3A) in order to further demonstrate the vital role of intercellular interaction during MSC therapy. The device was based on a highly hydrophobic glass surface where two identical chambers were separated by varied gap distances, with the narrowest around 500 μm and the widest around 1000 μm (Fig. 3B–D). Surface within the chambers was coated by gelatin for cell growth and the PDMS stamp was removed after successful cell attachment, leaving a corresponding cellular pattern on the glass (Fig. 3E). MSCs and mFBs could only communicate by a range of gap distances without any chance of direct contact. Likewise, α-SMA expression profile was utilized to assess the therapeutic potential of MSCs in this experimental setup. As reported previously, the paracrine activity was strictly dependent on the distance between cell populations *in vitro* (Hui and Bhatia, 2007). An obvious difference of the α-SMA fluorescence intensity was observed among mFBs which grew with different distances to MSCs (Fig. 3E a–c). Consistent to previous studies, the impact of MSCs on mFB phenotype was prominent within a short distance of separation (Fig. 3F) and the impact declined to comparable level as control (mono-cultured mFBs). However, comparison with the α-SMA intensity of directly co-cultured mFBs still showed an obvious difference, revealing that intercellular contact would be a dominant factor for MSCs’ therapeutic potential.

### Dynamic interaction between MSCs and mFBs

**Figure 4:**
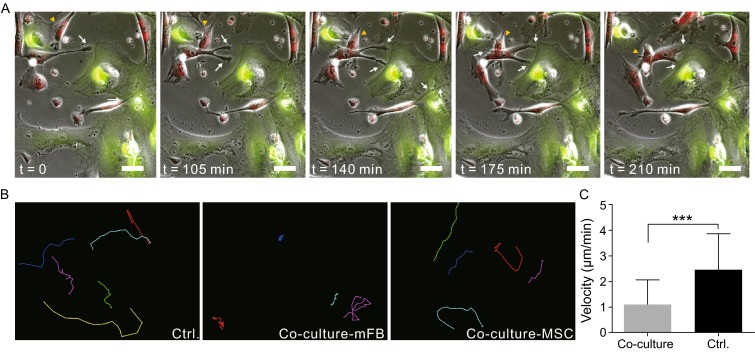
**Dynamic observation of direct MSC/mFB co-culture and motility analysis of mFBs**. (A) Time-lapse images of the co-culture. Cells were labelled with red (MSCs) or green (mFBs) CellTracker stains. Capture time points were indicated on each frame. White arrows highlighted the dynamic physical contacts between MSCs and mFBs. (B) Cell movement traces of mFBs in control group or co-cultures and those of MSCs in co-cultures. (C) Quantitative analysis of mFBs movement velocity between co-culture and mono-culture (control). Scale bar = 50 μm

In order to visualize the intercellular contacts, real-time interaction between MSCs and mFBs was monitored using time-lapse recording technology. Distinguishing the two populations was realized by pre-labelling MSCs and mFBs with dyes of distinct colors for live cell imaging. Obvious physical connections were observed between MSCs and mFBs (Fig. 4A, supplementary video 1), while similar connections were rare within the same populations (data not shown). ‘Tentacles’ (white arrows in Fig. 4A) stretching out of MSCs would target towards mFBs and gradually reach the cell membranes. The interaction would last for several hours followed by MSCs leaving and targeting other mFBs (black arrowhead in Fig. 4A). Strikingly, a prominent different mobility profile was observed for mFBs during co-culture. The cell movement traces were extracted from frames of time-lapse records and it was clear that mFBs in co-culture mostly resided in limited areas, and this was irrelevant to cell density as mono-cultured mFBs (control) with the same cell number could travel much more actively than co-cultured counterparts (Fig. 4B, supplementary video 2). However, the motility of MSCs was not affected by co-culture. As shown in previous study (Noiseux et al., 2006), transplanted MSCs at infarct border areas could travel or penetrate into the infarct zone, suggesting the migration and successful engraftment of MSCs into ischemic tissues, which was consistent with the observation here. Moreover, the movement velocity of cells was analyzed using the extracted traces and a great reduction of mFB motility was clearly shown in Fig. 4C.

### Directional migration property of mFBs during co-culture

**Figure 5:**
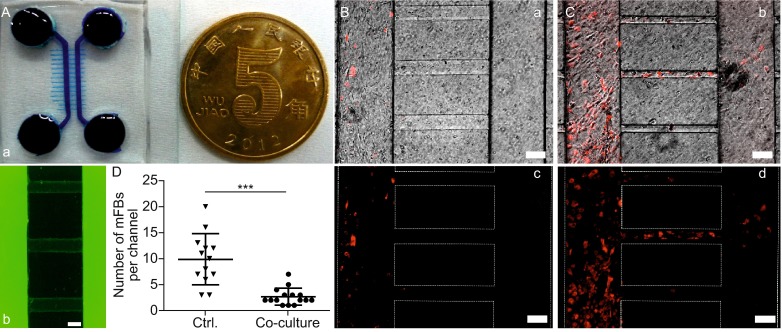
**Investigation of mFB migration using compartmentalized microfluidic device**. (A) Macro-size demonstration of the device compared with a 50 cent coin (25 mm diameter) (a). The chambers in the device were connected by micro-sized channels, indicated by AlexaFluor 488-conjugated IgG (b). (B and C) mFBs were seeded into the device either with MSCs (co-culture) or alone (control). mFBs were labelled by red CellTracker stain. Few mFBs migrated towards the other empty chamber in co-cultures (a, c), while cells would move to the other side across the channels without MSCs (b, d). Quantitative analysis of migrated mFBs was shown in (D). Scale bar = 200 μm

As to precisely assess the impact of MSC co-culture on mFBs’ motility, a well-accepted device for cell migration studies was fabricated (Fig. 5A a). The device was composed of PDMS and compartmentalized culture chambers were generated in a similar way to the device used in gap mode co-culture. Nonetheless, the two chambers were connected by micro-channels (Fig. 5A b), enabling cells to migrate from one side to the other. mFBs with or without MSCs were seeded into one chamber of the device, leaving the other side empty for observing migrated cells. After 48 h, many mFBs that were pre-labelled red were found in the empty chamber and within the micro-channels (Fig. 5C). Few red cells were discovered either in the channel or the opposite chamber in co-culture (Fig. 5B).

Though no inducible agent was added inside the empty chamber, cells would still move towards the opposite due to limited growth space. However, the proliferation of mFBs was mostly inhibited by MSCs as above investigated. Importantly, the expression of α-SMA, the major cytoskeletal actin that mFBs depend on to contract and migrate, was also proved to be greatly reduced due to the co-culture. Thus mFBs in co-culture could hardly migrate for long distance in the engineered system.

### Formation of cell-to-cell contacts during co-culture

**Figure 6:**
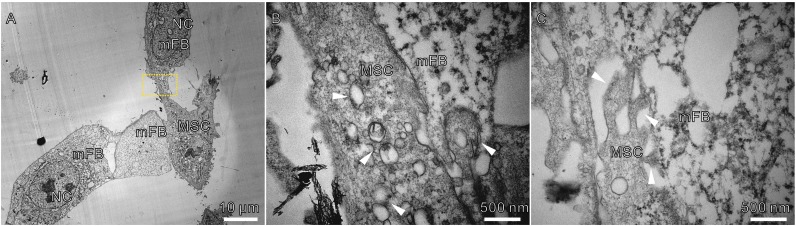
**Formation of cell-to-cell contacts during co-culture**. (A) Physical contacts formed between MSC and mFB during co-culture. (B and C) magnified views of boxed region in (A). Arrowheads highlighted the vesicles inside the cytoplasm of MSCs (B) or tunnels towards mFBs (C). NC: nucleus

Since cell staining is tricky for TEM analysis, we have to distinguish the two different cell populations by comparing the cellular morphology in mono-cultures. MSCs and mFBs could be distinguished clearly due to their unique membrane morphology and cytoplasmic composition under TEM (Figs. S2 and S3). TEM revealed that tight intercellular connections formed between MSCs and mFBs (Fig. 6A) and abundant vesicles were observed inside the cytoplasm of MSCs where connections were formed (Fig. 6B). Furthermore, tube-like structures were discovered as potential tunnels for vesicle transportation and other mass transferring towards mFBs (Fig. 6C). In addition, fiber-like structures could be obviously found both on membranes and within cytoplasm in mono-cultured mFBs (Fig. S3) while similar structures could rarely be found in co-cultured mFBs. These fibers were assumed to be α-SMA and the difference was consistent with the previous observation of reduced expression of α-SMA (Fig. 2A).

## CONCLUSION

In this study, we employed an *in vitro* interactive cell co-culture model to demonstrate that adipose-derived MSCs could ameliorate the key pathological characteristics of cardiac fibrosis mediated by mFBs mainly in a direct cell-to-cell contact manner, namely direct intercellular communication. Consistent with previous studies, we obtained the inhibitory effects of conditioned medium from MSCs on the viability and α-SMA expression of mFBs, however, the effects were more significant in the direct co-culture system. Hepatocyte growth factor (HGF), one of the known factors that reduce fibrosis in multiple organs, was demonstrated to be a major contributor for MSCs’ anti-fibrosis function. Moreover, using the engineered co-culture platform, the paracrine activity was found to be dependent on the distance between the two cell populations and be efficient at a narrow gap width (500 μm). Proper therapeutic benefits of MSCs to myocardial infarction should be based on the amelioration of fibrosis condition, in order to provide a favorable microenvironment for cell survival and factor diffusion. The study here, for the first time, elucidated that MSCs could exert better therapeutic effects by directly communicating with pathogenic cells (i.e. mFBs in cardiac fibrosis). We anticipated that this study could provide novel insight and more precise guidance for cell therapy based on MSCs, e.g. to introduce MSCs sufficiently close to the disease foci. Systematic investigations into anti-fibrosis molecules that were involved during the therapy process were necessary to provide better understanding of the underlying molecular mechanisms for the regenerative capacity of MSCs and could lead to establishment of novel therapeutic approaches.

## MATERIALS AND METHODS

### Materials

Polyethylene glycol (PEG, MW 258), 3-Trimethoxysilyl-propyl-methacrylate (TMSPMA), and Octadecyltrichlorosilane (OTS) were purchased from Sigma-Aldrich (St. Louis, MO). The photo initiator, 2-hydroxy-1-[4-(hydroxyethoxy)-phenyl]-2-methyl-1-pro-panone (Irgacure D2959), was purchased from Insight High Technology Co. LTD (China). CellTiter-Blue cell viability assay kit (alamar blue) was purchased from Promega (Wisconsin, USA).

### Cell culture

All animal experiments were approved by the Animal Ethics Committee of the Center of Biomedical Analysis, Tsinghua University. Cardiac fibroblasts were derived from hearts of Sprague-Dawley rats (male, 80–100 g) as previously described (Santiago et al., 2010) except some modifications (Zhao et al., 2014). Briefly, left ventricle tissue was excised, washed with HBSS (Wisent, Canada) and cut into pieces of ~1 mm^3^. Enzymatic digestion using Collagenase Type II (100 U, Gibco) and trypsin–EDTA (0.125%, Wisent, Canada) was employed to dissociate the tissue pieces, which were agitated at 37°C for 40~60 min. Supernatant was collected every 10 min into a conical tube and digestion was neutralized with 1/10 volume of fetal bovine serum (Wisent, Canada). Finally, the cells were centrifuged at 1,200 rpm for 5 min, resuspended in fresh medium (DMEM containing 10% FBS) and plated into 25 cm^2^ culture flasks. Non-adherent cells were discarded after 60 min. The rest of cells were propagated 4~5 passages, which would be induced spontaneously into mFBs as reported (Santiago et al., 2010).

MSCs were isolated from human adipose tissues obtained from patients undergoing liposuction operation as previously reported (Li et al., 2010). After isolation, the MSCs were expanded in growth medium (BIOWIT, China) and incubated at 37°C in a humidified environment containing 5% CO_2_. The isolated MSCs were positive for CD29, CD44, CD105 and Flk-1 while negative for CD31, CD34, CD45 and HLA-DR, as described earlier (Cao et al., 2005).

For direct co-culture of MSCs with mFBs, cells were mixed in ratios ranging from 1:0.01~1:1 (mFBs:MSCs) and plated in a density of 10,000/cm^2^ (Rahmat et al., 2013). Co-culture medium was composed by half of fibroblast medium (DMEM containing 10% FBS) and the other half of MSC growth medium. To prepare conditioned medium of adipose-derived MSCs, 90% confluent MSCs (less than passage 5) were washed with PBS and then exposed in fresh co-culture medium for 24 h (Yang et al., 2013). The medium was then collected and filtered through a 0.22 μm filter unit (Millipore) before use. All analyses were conducted 48 h post co-culture or treatment in conditioned medium.

### Cell viability analysis

Viability was assayed using CellTiter-Blue^®^ cell viability assay kit and performed as per manufacturer’s instructions. For viability assay, MSCs in the co-culture were inactivated by 10 μg/mL mitomycin C (Dalian Meilun Biology Technology Co., Ltd, China) for 3 h before being mixed with mFBs. Cells were plated in 48-well plate with constant number of mFBs and an equal number of inactivated MSCs used in the co-culture among each ratio were plated in individual wells for background elimination. Before the assay, culture medium was discarded. Alamar blue solution prepared in fresh medium was added to each well and incubated at 37°C. Fluorescence values were read by microplate reader (Molecular Devices, USA) with excitation at 560 nm and emission at 590 nm. All values were normalized to the mFB mono cultures.

Cell apoptosis detection kit (Beyotime, China) was used to verify that the mFBs were viable in co-culture. All procedures were accomplished as per the instructions. Cells treated with 500 μmol/L H_2_O_2_ for 2 h were used as positive control.

### Immunofluorescence staining

Samples were prepared by culturing cells on circular coverslips, then fixed with absolute methanol at −20°C for 10 min and blocked with 5% *w*/*v* bovine serum albumin (biotechnology grade, Wisent). Primary antibody against α-SMA (rabbit, Abcam) was diluted at 1:500 and incubated with samples overnight at 4°C. This was followed by 1 h incubation with Alexa Fluor 488 goat anti-rabbit IgG at room temperature. Hoechst 33324 (1:4000, Invitrogen) was used to stain cell nuclei. Fluorescence observation was performed on Nikon Eclipse Ti-S microscope (Nikon, Japan). α-SMA fluorescence intensity was analyzed using ImageJ software (NIH, USA) according to software manual. Briefly, all the fluorescent images were taken with identical optical settings (i.e. exposure time and binning numbers). Images were then imported into ImageJ and more than 3 different ROIs in the image were selected for pixel intensity quantification.

### Collagen staining

Sirius Red collagen detection kit (Chondrex, Inc., USA) was used to quantify the amount of collagen as per manufacturer’s instructions. Similarly, an equal number of inactivated MSCs used in the co-culture among each ratio were plated in individual wells for background elimination. Briefly, samples were fixed and incubated with Sirius Red solutions for 30 min at room temperature, and eluted using extraction buffer provided in the kit. The absorbance of the extracted solution was read at 540 nm by microplate reader.

### Western blotting

Total proteins were extracted from cultured cells using RIPA lysing buffer (Beyotime, China), according to the manufacturer’s protocol. Proteins were separated by 10%–12% SDS/PAGE gels and transferred to PVDF membrane (Millipore), which was then blocked by 5% (*w*/*v*) nonfat dry milk in TBS-Tween (0.2%) for 1 h. Membranes were probed with rabbit anti-rat α-SMA (1:400, Abcam) overnight at 4°C. After several washes in TBS-Tween, membranes were incubated with goat anti-rabbit HRP-conjugated secondary antibody (1:2000, ZSGB-BIO, China). The subsequent visualization was performed using SuperSignal West Pico Chemiluminescent Substrate (Thermo) by the ChemiDoc XRS^+^ with image Lab software (Bio-Rad).

### Collagen contraction assay

Collagen gel lattice contraction assay was performed to measure contractility of mFBs. The collagen lattice was prepared by combining 20 μL of cold collagen solution (BD, USA), 80 μL co-culture medium and 0.46 μL of 0.1 mol/L sodium hydroxide solution on ice. mFBs were harvested from mono-culture, counted and resuspended in pre-mixed cold collagen solution at a density of 1 × 10^6^ cells/mL. For co-culture group, MSCs and mFBs were mixed equally to reach the same density. As to dispel the impact of mFBs’ number on gel contraction, control groups were prepared by encapsulating mFBs inside gels with an equal or half of the total cell number in co-culture. Then cell-collagen mixture was pipetted into 48-well plates (100 μL/well) and incubated at 37°C for 30 min to polymerize the collagen lattices. The plates were pre-coated with 5% bovine serum albumin (biotechnology grade, Wisent, Canada) in order to release the gels from the plates. After 30 min, 200 μL co-culture medium were added to each well. To assess the influence of anti-fibrosis cytokine, antibody against HGF (sc-13087, Santa Cruz) was dissolved in collagen solution (1:100) to block the factors secreted by MSCs, while HGF (R&D) was dissolved in collagen solution (20 ng/mL) to disturb normal contractility of mFBs. A digital camera was used to take pictures of the gel lattices after 18 h. The area of the lattices was analyzed using ImageJ software (NIH, USA). The extent of contraction was calculated by:

$${\text{extent}}\, = \,\frac{well\,area\, - \,gel\,area}{well\,area} \times \,100\%$$

### Time-lapse imaging

Time-lapse recording was performed to monitor the dynamic interaction between MSCs and mFBs. Culture plate was mounted in a 5% CO_2_ filled incubation chamber (Nikon, Japan). Cells were labeled with CellTracker (green for mFB and red for MSCs, Invitrogen) before imaging. Pictures were taken at 35 min interval using inverted Fluorescent Microscope (Ti-U series, Nikon, Japan) and cellular movement tracking was realized using ImageJ software equipped with an open access manual tracking plugin.

### Fabrication of compartmentalized microfluidic devices

The device was fabricated using PDMS stamps by conventional soft-lithography technique. The molds were fabricated using UV cross-linkable polyethylene glycol (PEG, Mw = 258, Sigma) through pre-designed photomask. Briefly, 1 wt% photo initiator (Irgacure D2959) was dissolved in PEG258 solution, which was then pipetted onto 3-(trimethoxysilyl)propyl methacrylate (TMSPMA) coated glass slides and exposed to UV light (OmniCure SERIES 1500, 27.6 mW/cm^2^, Canada) for 18~25 s. Then, the patterned molds were immersed into ethanol to remove any unreacted precursors for 5 min and air dried. Afterwards, PDMS prepolymer solution was poured onto the molds and polymerized at 70°C for 8 h.

Device for investigating directional cell migration was composed of two isolated chambers (2 cm long 150 μm high) interconnected by narrow channels (1 mm long 0 μm high). Device for investigating gap mode co-culture was composed of two separated chambers in the same size as above mentioned despite that they were separated by cell-repellent gaps in a range of distances.

For cell seeding in migration device, PDMS was removed from the molds and stamped on clean glass slides through thermal binding after 2 min of plasma treatment. The slides were then functionalized with 0.1% gelatin solution for cell adherence. Cells were seeded into one chamber at a density of 1 × 10^4^ cells/cm^2^. To prevent cell leakage, the interconnected micro-channel was filled with 2 mg/mL collagen gel prior to cell seeding.

For gap mode co-culture, PDMS stamp was pressed onto OTS-treated glass slides which would provide a cell-repellent surface between separated chambers, followed by plasma treatment that enabled protein adsorption in cell growing chambers. MSCs and mFBs were seeded into each chamber and allowed to adhere before stamp removal, leaving separated cell patterns on the slide. Co-culture medium was then added on top of the slide, covering both patterns and incubated for 48 h.

### Transmission electron microscopy (TEM)

For the TEM analysis, cells were immersed in 2.5% glutaraldehyde (VASE, China), fixed in 1% OsO_4_, dehydrated using 70% EtOH containing 2% uranyl acetate and embedded in Epon 812 (Fluka, USA). After Epon polymerized, the samples were removed from the culture dish and cut into ultra-thin (7 μm) sections using a Leica EM UC6. The sections were stained with lead citrate and examined using H-7650B microscope (Hitachi, Japan).

### Statistical analysis

Quantitative data were plotted as the mean ± standard deviation. Statistical analysis was performed using Student’s unpaired two-way *t*-tests and ANOVA analysis. Differences were considered to be statistically significant when *P* < 0.05.

## Electronic supplementary material

Supplementary material 1 (PDF 427 kb)

Supplementary material 2 (MOV 4120 kb)

Supplementary material 3 (MOV 984 kb)

## ABBREVIATIONS

α-SMA: alpha-smooth muscle actin
CVD: cardiovascular disease
HGF: hepatocyte growth factor
mFBs: myofibroblasts
MMPs: matrix metalloproteinases
MRI: magnetic resonance imaging
MSCs: mesenchymal stem cells
OTS: octadecyltrichlorosilane
PEG: polyethylene glycol
TMSPMA: 3-trimethoxysilyl-propyl-methacrylate
TGF-β1: transforming growth factor-beta 1
