# Supplementary material for: Direct intercellular communications dominate the interaction between adipose-derived MSCs and myofibroblasts against cardiac fibrosis
Source: Protein Cell. 2015 Aug 14;6(10):735–45. doi: 10.1007/s13238-015-0196-7 (PMC4598323; doi:10.1007/s13238-015-0196-7)
Supplement: Supplementary file 1 — Supplementary material 1 (PDF 427 kb) [file 13238_2015_196_MOESM1_ESM.pdf]

Titles and legends to Supplementary figures

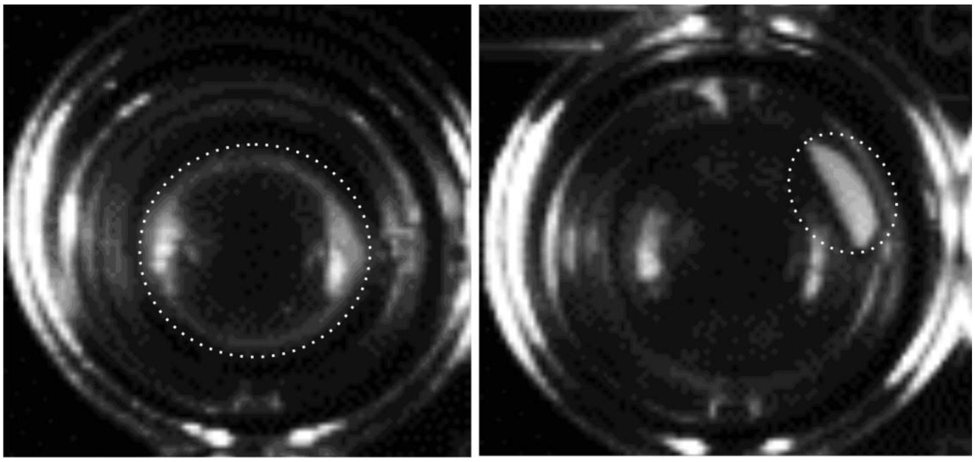

Supplementary figure 1. **Contractility of MSCs in mono-culture and co-culture**
Left: contraction of co-cultured cells; right: contraction of mono-cultured MSCs

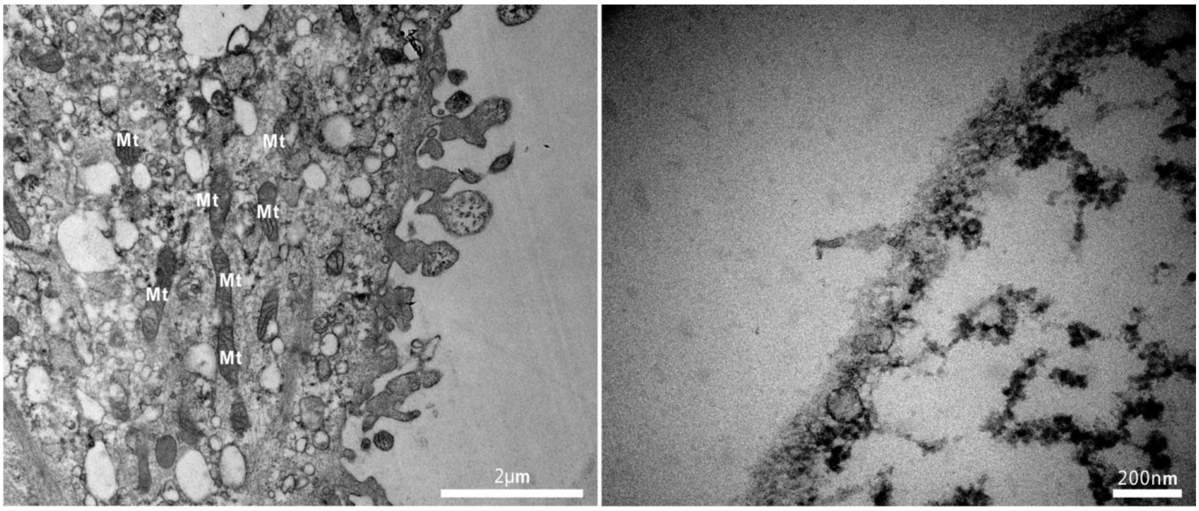

Supplementary figure 2. **Morphological differences of MSCs and mFBs under TEM**
Left: membrane morphology of MSCs. Mt: mitochondria within the cytoplasm of cells;
Right: membrane morphology of mFBs and sparse cytoplasm

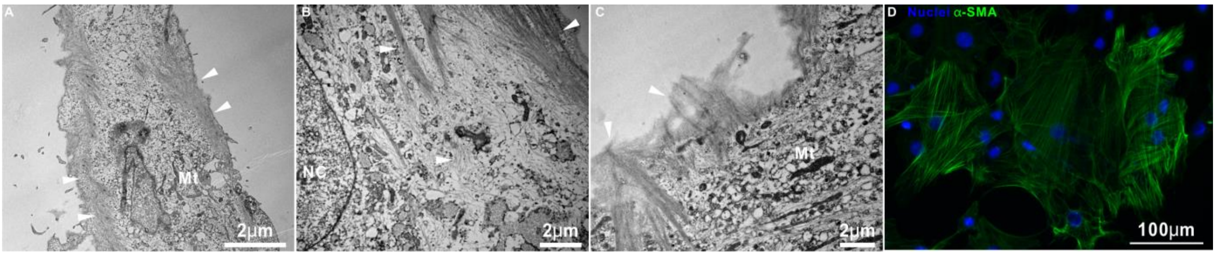

Supplementary figure 3. **Actin fibers of mFBs in mono-culture**
(A-C) TEM images of mono-cultured mFBs. Arrowheads highlighted the fiber structures. Mt: mitochondria; NC: nucleus (D) Immunofluorescence staining of α-SMA in mono-cultured mFBs, indicating the resemblance between the fiber structures observed by TEM and by staining.
